# Comparison of weight change between face-to-face and digital delivery of the English National Health service diabetes prevention programme: An exploratory non-inferiority study with imputation of plausible weight outcomes

**DOI:** 10.1016/j.pmedr.2023.102161

**Published:** 2023-02-20

**Authors:** Antonia M. Marsden, Mark Hann, Emma Barron, Jamie Ross, Jonathan Valabhji, Elizabeth Murray, Sarah Cotterill

**Affiliations:** a Centre for Biostatistics, School of Health Sciences, University of Manchester, Oxford Road, Manchester M13 9PL, UK; b Centre for Primary Care and Health Services Research, School of Health Sciences, University of Manchester, Oxford Road, Manchester M13 9PL, UK; c NHS England, London SE1 6LH, UK; d Centre for Primary Care, Wolfson Institute of Population Health Science, Barts and The London School of Medicine and Dentistry, Queen Mary University of London, London E1 2AD, UK; e Research Department of Primary Care and Population Health, University College London, Royal Free Campus, Pond Street, London NW3 2PF, UK; f Imperial College Healthcare NHS Trust, The Bays, S Wharf Rd, London W2 1NY, UK; g Imperial College London, Exhibition Rd, South Kensington, London SW7 2BX, UK

**Keywords:** (BMI), Body Mass Index, CCG, Clinical Commissioning Group, CI, Confidence interval, DIPLOMA, Diabetes Prevention – Long Term Multimethod Assessment, FPG, Fasting Blood Glucose (a test for diagnosing diabetes and the risk of diabetes), HbA1c, Haemoglobin A1c (a test for diagnosing diabetes and the risk of diabetes), IMD, Index of Multiple deprivation, NHS DPP, National Health Service Diabetes Prevention Programme, STP, Sustainability and Transformation Partnership, Weight loss, Diabetes mellitus, Type 2, National health programs, eHealth: Telemedicine, Diet, healthy, Preventive health services, Self-management, Non-inferiority, Cohort studies, Method for dealing with missing data

## Abstract

Worldwide evidence suggests face-to-face diabetes prevention programmes are effective in preventing and delaying the onset of type 2 diabetes by encouraging behaviour change towards weight loss, healthy eating, and increased exercise. There is an absence of evidence on whether digital delivery is as effective as face-to-face. During 2017–18 patients in England were offered the National Health Service Diabetes Prevention Programme as group-based face-to-face delivery, digital delivery (‘digital-only’) or a choice between digital and face-to-face (‘digital-choice’). The contemporaneous delivery allowed for a robust non-inferiority study, comparing face-to-face with digital only and digital choice cohorts.

Changes in weight at 6 months were missing for around half of participants. Here we take a novel approach, estimating the average effect in all 65,741 individuals who enrolled in the programme, by making a range of plausible assumptions about weight change in individuals who did not provide outcome data. The benefit of this approach is that it includes everyone who enrolled in the programme, not restricted to those who completed. We analysed the data using multiple linear regression models.

Under all scenarios explored, enrolment in the digital diabetes prevention programme was associated with clinically significant reductions in weight which were at least equivalent to weight loss in the face-to-face programme. Digital services can be just as effective as face-to-face in delivering a population-based approach to the prevention of type 2 diabetes. Imputation of plausible outcomes is a feasible methodological approach, suitable for analysis of routine data in settings where outcomes are missing for non-attenders.

## 1 Introduction

Diabetes is a chronic health condition associated with numerous adverse outcomes including microvascular disease, cardiovascular disease, and premature death. Both the incidence and prevalence of Type 2 diabetes is increasing globally, and prevention has become a major international public health objective (Bergman et al., 2012, Saeedi et al., 2019, World Health Organization, 2016). Many countries have initiated diabetes prevention programmes which target those at highest risk of type 2 diabetes and encourage change in diet and exercise behaviours, with the objective of delaying or preventing onset of disease. Evidence from around the world suggests that face-to-face, group-based diabetes prevention programmes can be effective in reducing the incidence of diabetes (Ashra et al., 2015, Galaviz et al., 2018).

Digital behaviour change interventions have been shown to be as effective as in-person interventions in other healthcare settings (Luo et al., 2020), including programmes for weight loss among people who are overweight (Beleigoli et al., 2019). However, there is less strong evidence supporting digital delivery for diabetes prevention (Bian et al., 2017, Grock et al., 2017, Joiner et al., 2017, Van Rhoon et al., 2020).

The English National Health Service Diabetes Prevention Programme (NHS DPP), “Healthier You”, was based on international evidence, encouraging healthy eating, weight loss and increased exercise in people at high risk of developing Type 2 diabetes, according to national guidelines (HbA1c 42–47 mmol/mol [6.0–6.4%] or fasting plasma glucose (FPG, 5.5–6.9 mmol/L) (Hawkes et al., 2020a, Hawkes et al., 2020b, National Institute for Health and Care Excellence (NICE), 2012). The NHS offered patients face-to-face service delivery, for which there was stronger evidence, and it was rolled out in stages across England between 2016 and 2018. There was an NHS experimental pilot in 2017–18 which offered a digital service to patients in selected areas of England (Ross et al., 2022). The digital service was introduced in two ways: (i) in some localities face-to-face delivery was not yet available and patients were only offered a digital service (digital-only) (ii) in other localities, patients were offered a choice between digital and face-to-face delivery (digital choice). The face-to-face and digital services were commissioned by the same NHS team from external providers, using comparable service specifications and offering similar behaviour change and self-management content. Previous studies have reported that mean weight loss at 6 months was 3.2 kg [95% confidence interval (CI): 3.1, 3.3] in the face-to-face service (Marsden et al., 2022a) and 3.5 kg [95% CI: 3.3, 3.7) in the digital service (Ross et al., 2022).

The contemporaneous delivery of face-to-face and digital versions of the same programme content allowed for a robust non-inferiority observational study, comparing face-to-face with the digital only and digital choice cohorts, with adjustment for differences between the participating populations. We have reported elsewhere that weight change on the digital pilot was non-inferior to face-to-face at 6 months. Mean weight loss among those who were offered a choice and chose digital was higher than face to face (difference in weight change: −1.165 kg [95% CI: −1.841, −0.489], and among those with no choice it was similar (−0.284 kg [95% CI: −0.712, 0.144])) (Marsden et al., 2022b).

The primary analysis in our previous study was a complete case analysis, comparing change in weight only in those patients who provided weight measures at 6 and 12 months; there was a substantial amount of missing data for people who did not attend to have their weight measured (Marsden et al., 2022b). We considered a multiple imputation approach to be unsuitable because the weights were likely missing ‘not at random’. That is, the probability of a weight measure being missing, even after accounting for covariates, is dependent on the value of the missing weight (Austin et al., 2021). Most of the missing data was from people who had stopped participating in the programme, and it seemed implausible to impute weights for non-participants using data from those who continued to participate.

The aim of the current paper is to estimate the average effect in all individuals who enrolled in the digital or face-to-face cohorts, by making plausible assumptions about the change in weight of individuals who did not provide outcome data. We considered a range of plausible changes in weight in both the face-to-face and digital groups, using a non-inferiority approach, and analysed the data using multiple linear regression models to see what impact this has on the overall conclusion. This was a pre-specified exploratory analysis. The benefit of this approach over the previous analysis is that it includes everyone who enrolled in the programme, rather than being restricted to those who completed the programme and provided outcome data. Unlike multiple imputation and Inverse Probability Weighting, this approach incorporates the fact that the outcome data are missing not at random. It is also a simple and intuitive approach. The study was pre-registered on the Open Science Framework 14 July 2021 (Marsden, 2021).

## 2 Material and methods

### 2.1 Research design

The design was a retrospective observational cohort study, using patient-level data collected by NHS DPP service providers. Details of the diabetes prevention programme and the population inclusion criteria are fully reported elsewhere (Hawkes et al., 2020a, Ross et al., 2022). In summary, the face-to-face service offered a one-to-one initial assessment followed by at least thirteen group sessions, which delivered behaviour change content related to diet, weight loss and increased exercise with regular group education and exercise sessions (Hawkes et al., 2020a, NHS England, 2022). The digital service offered similar content (Ross et al., 2022). There were two digital cohorts in the analysis. The ‘digital only’ cohort were those who lived in areas without any face-to-face DPP, and so their only option was digital. The ‘digital choice’ cohort lived in areas where both digital and face-to face services were operating and they were offered a choice. All participants were referred from primary care. NHS England published a service specification which outlined what the broad content of the programme should look like; the services were delivered by several independent providers, and specific content varied across the providers (NHS England, 2016).

The pre-specified non-inferiority margin for change in weight at 6 months was determined by the NHS DPP Expert Reference Group (NHS England, 2022) as 1 kg. For example, if average change in weight via face-to-face delivery was no greater than 1 kg more than via digital delivery, digital was deemed non-inferior.

### 2.2 Population

Eligible patients were those with non-diabetic hyperglycaemia who were referred to the digital or face-to-face NHS DPP in 2017–18 and who either attended a first session of the face-to-face service or registered for the digital service, and provided a baseline weight measure.

### 2.3 Data collection

Data was collected by service providers and compiled by NHS England. This process is described fully elsewhere (Marsden et al., 2022b). In summary, personal characteristics were recorded at baseline. These included age at referral, sex, ethnicity, socioeconomic deprivation (defined by the English Index of Multiple deprivation (IMD) 2015 associated with the individual’s local area, grouped into quintiles), HbA1c (a widely used measure of blood glucose, used to assess diabetes risk) in mmol/mol and body mass index (BMI) in kg/m^2^. The area in which the participant resided was described via health-administration geographical areas: Clinical Commissioning Group (CCG) and Sustainability and Transformation Partnership (STP). Each STP commissioned a single service provider and CCGs managed the local implementation of referrals from General Practice. Weight was recorded at group sessions (face-to-face), and in General Practice, pharmacies or at home (digital), using Wi-Fi enabled pre-calibrated equipment supplied by the provider (which automatically uploaded the recorded weight). There were no self-reported weight measures. The same mode of measurement was used for a participant’s baseline and follow-up observations. We defined baseline weight as that measured at the first intervention session attended (face-to-face) or registration (digital) and 6-month weight as that closest to 6 months after baseline (and within 4–8 months).

Weight in face-to-face delivery was collected if participants continued to attend sessions. Hence, changes in weight were missing for anyone who stopped attending before the 6-month weight was collected. In the digital pilot, all individuals who registered were invited to provide 6-month data, regardless of whether they were still enrolled. Changes in weight were missing for 29,080 (47%) in the face-to-face cohort, 753 (42%) in the digital only cohort and 1309 (62%) in the digital choice cohort.

### 2.4 Assumed plausible outcomes

Weight at 6 months was estimated in one of four ways, depending on the data available:

#### 2.4.1 Observed values

For those who provided weight outcome data, their observed values were used.

#### 2.4.2 Regression mean imputation

For those in the face-to-face cohort who were recorded as still participating in the programme at 6 months, but where a weight measure was not recorded, regression mean imputation was used to impute an estimated outcome. Our justification for this was that it is reasonable to assume that their outcomes, if they had been collected, would be like other participants for whom a 6-month weight had been measured.

#### 2.4.3 Assumed plausible outcomes

For all individuals who did not provide outcome data in the digital cohort, and individuals in the face-to-face cohort who did not provide outcome data and who were not participating in the programme at the time, we imputed a range of plausible assumptions. These were based on clinical opinion, and the thinking underpinning the assumptions was that weight gain is the expected natural progression in untreated people at risk of diabetes, and weight loss could be due to being informed of high risk of T2DM and undertaking behaviour change on their own. The following changes in weight from the first intervention session attended to 6 months were assumed:

1. No change
2. 0.5 kg increase
3. 1 kg increase
4. 0.5 kg decrease
5. 1 kg decrease

Since some individuals in the face-to-face cohort who did not provide a 6 m weight did provide one or more weight measures after baseline, scenarios 6–10 incorporate these by assuming the following changes in weight (excluding measures collected within one month of baseline, which we thought was too close to baseline):

6. Change from baseline to last observed weight (if available),
  No change otherwise.
7. Change from baseline to last observed weight (if available),
  0.5 kg increase otherwise.
8. Change from baseline to last observed weight (if available),
  1 kg increase otherwise.
9. Change from baseline to last observed weight (if available),
  0.5 kg decrease otherwise.
10. Change from baseline to last observed weight (if available),
  1 kg decrease otherwise.

### 2.5 Statistical analyses

Mixed effects linear regression modelling was used to compare change in weight from baseline to 6 months between the face-to-face cohort and each digital cohort separately. An indicator variable (face-to-face/digital-only/digital-choice) was included to convey the estimated adjusted difference in mean change in weight at 6 months between face-to-face and each digital group, with face-to-face as the reference group. We chose a non-inferiority over an equivalence design because our purpose was to estimate whether digital delivery was not inferior (equivalent or possibly superior) to face-to-face, unlike an equivalence study would have considered only whether digital delivery was strictly equivalent (Walker and Nowacki, 2011)*.* Non-inferiority was inferred if the upper bound of the 95% confidence interval for this adjusted difference was lower than the 1 kg non-inferiority limit. The model adjusted for the timing of the outcome measure (months from baseline) as fixed effects and CCG nested within STP as random effects to account for variation across sites. The timing of the weight measure was set to 6 months where this outcome was originally missing.

## 3 Results

A summary of baseline characteristics in those with and without a missing 6-month weight is shown in Table 1. In all delivery modes, the distribution of sex was similar in the missing and non-missing groups. The non-missing groups in face-to-face and digital only were, on average, older, had a lower baseline weight and BMI, and had a higher proportion of individuals with White ethnicity than the missing groups. In the face-to-face and digital choice delivery modes, there was a higher proportion of individuals from the most deprived quintile in the missing group compared to the non-missing group. The mean baseline HbA1c was higher in those with a missing 6-month weight value in the digital-choice group.

**Table 1:** Baseline characteristics of adults enrolled in NHS DPP 2017–18, comparing those with and without a missing 6-month weight value.

	Face-to-face		Digital only		Digital choice	
	Not missing (n=32744)	Missing (n=29122*)	Not missing (n=1025)	Missing (n=753)	Not missing (n=833)	Missing (n=1324)
**Sex,** N(%)						
Male	14865 (45.5)	13370 (46.0)	475 (46.4)	361 (48.1)	414 (50.0)	645 (49.5)
Female	17810 (54.5)	15691 (54.0)	548 (53.6)	390 (51.9)	414 (50.0)	658 (50.5)
p-value^a^	0.201		0.549		0.822	

**Age at referral**						
Mean (SD)	66.6 (10.4)	63.3 (12.9)	59.6 (12.2)	56.0 (12.0)	59.5 (11.5)	60.3 (14.6)
Median (IQR)	68 (61, 74)	65 (55, 73)	61 (52, 69)	57 (48, 65)	61 (52, 68)	61 (51, 71)
p-value^a^	<0.001		<0.001		0.191	

**Ethnicity,** N(%)						
White	25543 (84.6)	19217 (73.0)	781 (78.7)	502 (70.5)	642 (83.8)	699 (84.7)
Mixed	459 (1.5)	591 (2.2)	14 (1.41)	14 (1.94)	19 (2.48)	18 (2.18)
Asian	2328 (7.7)	4155 (15.8)	141 (14.2)	142 (19.9)	77 (10.1)	72 (8.73)
Black	1444 (4.78)	1845 (7.01)	55 (5.54)	53 (7.44)	23 (3.00)	33 (4.00)
Other	412 (1.36)	519 (1.97)	2 (0.20)	1 (0.14)	5 (0.65)	3 (0.36)
p-value^a^	<0.001		0.004		0.606	

**IMD Quintile**^b^**,** N(%)						
1 (Most deprived)	4168 (12.8)	6287 (21.6)	150 (14.7)	110 (14.6)	205 (24.7)	618 (48.3)
2	5507 (16.9)	5413 (18.6)	227 (22.2)	196 (26.1)	117 (14.1)	223 (17.4)
3	6533 (20.0)	5396 (18.6)	309 (30.2)	198 (26.3)	108 (13.1)	167 (13.1)
4	7479 (22.9)	5591 (19.2)	207 (20.2)	156 (20.7)	183 (22.1)	147 (11.5)
5 (Least deprived)	8990 (27.5)	6380 (22.0)	131 (12.8)	92 (12.2)	217 (26.1)	124 (9.70)
p-value^a^	<0.001		0.27		<0.001	

**Weight in kg at baseline**						
Mean (SD)	83.23 (18.13)	85.18 (19.38)	87.43 (19.15)	89.56 (21.02)	86.98 (19.04)	86.10 (21.22)
Median (IQR)	81.2 (70.4, 93.6)	82.8 (71.6, 96.0)	85.0 (73.8, 99)	88.0 (74.0, 101.5)	85.0 (74.0, 98.0)	83.9 (71.1, 97.2)
p-value^a^	<0.001		0.026		0.332	

**Body Mass Index (BMI) at baseline**						
Mean (SD)	29.95 (5.70)	30.75 (6.20)	31.06 (5.97)	31.72 (6.58)	30.46 (5.99)	30.76 (6.71)
Median (IQR)	29.1 (26.0, 32.9)	29.8 (26.5, 34.0)	30.0 (26.6, 34.4)	30.5 (27.1, 35.3)	29.3 (26.3, 33.4)	29.6 (26.3, 34.0)
N(%)						
Normal weight	5865 (18.1)	4524 (15.8)	122 (11.9)	97 (12.9)	122 (14.7)	224 (17.1)
Overweight	12503 (38.5)	10212 (35.7)	390 (38.1)	241 (32.1)	329 (39.6)	458 (35.0)
Obese	12352 (38.0)	11616 (40.6)	427 (41.7)	332 (44.2)	321 (38.7)	498 (38.0)
Severely obese	1759 (5.4)	2287 (8.0)	86 (8.39)	81 (10.8)	58 (6.99)	129 (9.85)
p-value^a^	<0.001		0.027		0.303	

**HbA1c**^d^**at baseline**						
Mean (SD)	40.7 (4.0)	40.8 (4.2)	43.9 (2.0)	43.9 (1.9)	43.1 (2.4)	43.6 (1.8)
Median (IQR)	41 (38, 43)	41 (38, 43)	44 (42, 45)	44 (43, 45)	43 (42, 45)	43 (42, 45)
N(%)						
Normal range	10184 (55.6)	7571 (55.1)	29 (2.83)	18 (2.40)	71 (8.6)	38 (2.92)
NDH^C^	7579 (41.4)	5736 (41.8)	984 (96.2)	714 (95.3)	757 (91.2)	1261 (96.9)
T2DM	560 (3.06)	426 (3.10)	10 (0.98)	17 (2.27)	2 (0.24)	3 (0.15)
p-value^a^	0.663		0.655		<0.001	

*Explanation for why this is 29,122 instead of the 29,080 reported in the results section: there are 42 people who had a missing 6 month weight value and who were still participating in the programme at the 6 month time point, but regression mean imputation could not be performed as they had a missing ethnicity value.

a p-value from a two-sample *t*-test test for age at referral, weight at baseline, BMI at baseline and HbA1c at baseline, and a chi-square test for sex, ethnicity and IMD quintile.

b Index of multiple deprivation (IMD) score for an individual’s small-area of residence, grouped using quintiles.

C Nondiabetic hyperglycaemia (NDH) describes adults at high risk of developing type 2 diabetes (T2DM, defined as having HbA1c 42–47 mmol/mol [6.0–6.4%] or fasting plasma glucose (FPG, 5.5–6.9 mmol/L) Barron et al., 2018, Howarth et al., 2020, Valabhji et al., 2020).

d HbA1c is a blood glucose test, widely used to assess diabetes risk.

By assuming an outcome for those for whom this was missing, cohort-specific sample sizes increased: from 32,744 to 61,824 for the face-to-face cohort (29080 were replaced (47%)), from 1025 to 1778 for the digital-only cohort (753 were replaced (42%)) and from 830 to 2139 for the digital choice cohort (1309 were replaced (62%)). Baseline characteristics of the sample are reported elsewhere (Marsden et al., 2022b). In summary, in comparison to the face-to-face-cohort, the digital only cohort was younger, with a slightly larger proportion of ethnic minorities, fewer people from the most and least deprived areas and higher baseline weights; the digital choice cohort had a slightly higher proportion of males, was younger, had a slightly lower proportion of ethnic minorities, greater numbers from the most deprived areas and higher baseline weights.

Table 2 shows the raw mean change in weight (with a 95% confidence interval) across the three cohorts under the various assumptions about the missing outcome data. Under all assumptions considered, in all three cohorts, patients lost weight between baseline and 6 months. The estimated weight loss ranged between 1.07 kg and 2.06 kg (face-to-face), 1.30 kg and 2.18 kg (digital only) and 0.86 kg and 2.08 kg (digital choice) depending on the assumptions made (Table 2). Mean weight loss as a % of mean baseline weight ranged between 1.32% and 2.54% (face-to-face), 1.52% to 2.47% (digital only) and 0.98% to 2.36% (digital choice).

**Table 2:** Mean change in weight at 6 months of adults enrolled in NHS DPP 2017–18 in the face-to-face cohort and both digital cohorts under missing outcome data assumptions.

Mean change (kg) (95% CI)	Assumptions about weight loss in those who did not provide 6-month weigh measures	Face-to-face	Digital only	Digital choice
N		32,744	1025	830
	Complete case analysis(Marsden et al., 2022b)	−2.85 (−2.89, −2.81)	−3.05 (−3.38, −2.73)	−3.79 (−4.16, −3.43)

N		61,824	1778	2139
	(1) no change a	−1.53 (−1.55, −1.50)	−1.76 (−1.96, −1.56)	−1.47 (−1.63, −1.31)
	(2) 0.5 kg increase a	−1.30 (−1.32, −1.27)	−1.54 (−1.75, −1.35)	−1.17 (−1.33, −1.00)
	(3) 1 kg increase a	−1.07 (−1.09, −1.04)	−1.34 (−1.54, −1.13)	−0.86 (−1.03, −0.69)
	(4) 0.5 kg decrease a	−1.76 (−1.79, −1.74)	−1.97 (−2.17, −1.78)	−1.78 (−1.94, −1.62)
	(5) 1 kg decrease a	−1.99 (−2.01, −1.97)	−2.18 (−2.38, −1.99)	−2.08 (−2.24, −1.93)
	(6) Latest observation or no change a	−1.87 (−1.89, −1.84)	−1.76 (−1.96, −1.56)	−1.47 (−1.63, −1.31)
	(7) Latest observation or 0.5 kg increase a	−1.62 (−1.65, −1.60)	−1.54 (−1.75, −1.35)	−1.17 (−1.33, −1.00)
	(8) Latest observation or 1 kg increase a	−1.48 (−1.50, −1.45)	−1.34 (−1.54, −1.13)	−0.86 (−1.03, −0.69)
	(9) Latest observation or 0.5 kg decrease a	−1.91 (−1.94, −1.89)	−1.97 (−2.17, −1.78)	−1.78 (−1.94, −1.62)
	(10) Latest observation or 1 kg increase a	−2.06 (−2.08, −2.03)	−2.18 (−2.38, −1.99)	−2.08 (−2.24, −1.93)

a Where individuals had no 6-month weight measure recorded, weights collected 1–4 months after baseline were used, but if not observed, the plausible values were imputed.

The average change in weight was lower than the observed change in the main analysis, as was expected from an analysis which includes patients who did not complete the programme and assumes their weight loss was lower than the average of completers. Under the most extreme positive assumption, that those with a missing outcome lost 1 kg in weight (assumption 5), the mean changes were −1.99 (95% CI: −2.01, −1.97), −2.18 (95% CI: −2.38, −1.99) and −2.08 (95% CI: −2.24, −1.93) in the face-to-face, digital only and digital choice cohorts respectively (Table 2).

Table 3, Table 4 show the output from the linear mixed model analyses comparing change in weight at 6 months between the face-to-face and each of the two digital cohorts.

**Table 3:** Regression analyses comparing change in weight among adults enrolled in NHS DPP 2017–18 (baseline to 6 months) between the face-to-face cohort and the digital only cohort under missing outcome data assumptions.

Assumption	N	Difference in weight change between digital only and face-to-face	95% CI	p-value
(1) No change a	58,000	−0.368	(−0.711, −0.025)	0.036
(2) 0.5 kg increase a	58,000	−0.427	(−0.783, −0.070)	0.019
(3) 1 kg increase a	58,000	−0.486	(−0.857, −0.115)	0.010
(4) 0.5 kg decrease a	58,000	−0.309	(−0.639, 0.022)	0.067
(5) 1 kg decrease a	58,000	−0.250	(−0.569, −0.069)	<0.001
(6) Latest observation or no change a	58,000	−0.014	(0.367, 0.338)	0.936
(7) Latest observation or 0.5 kg increase a	58,000	−0.010	(−0.378, 0.359)	0.959
(8) Latest observation or 1 kg increase a	58,000	0.057	(−0.326, 0.440)	0.772
(9) Latest observation or 0.5 kg decrease a	58,000	−0.138	(−0.480, 0.205)	0.430
(10) Latest observation or 1 kg increase a	58,000	−0.197	(−0.529, 0.134)	0.242

a Model adjusts for age at referral, sex, ethnicity (white/mixed/black/Asian/other), Index of Multiple Deprivation (IMD) quintile, time since baseline (in months) as fixed effects and Clinical Commissioning Group (CCG) nested within Sustainability and Transformation Partnership (STP) as random effects.

**Table 4:** Regression analyses comparing change in weight among adults enrolled in NHS DPP 2017–18 (from baseline to 6 months) between the face-to-face cohort and the digital choice cohort under missing outcome data assumptions.

Assumption	N	Difference in weight change between digital choice and face-to-face	95% CI	p-value
(1) No change a	57,890	−1.006	(−1.366, −0.647)	<0.001
(2) 0.5 kg increase a	57,890	−1.070	(−1.444, −0.697)	<0.001
(3) 1 kg increase a	57,890	−1.136	(−1.525, −0.747)	<0.001
(4) 0.5 kg decrease a	57,890	−0.943	(−1.289, −0.597)	<0.001
(5) 1 kg decrease a	57,890	−0.882	(−1.216, −0.549)	<0.001
(6) Latest observation or no change a	57,890	−0.678	(−1.049, −0.306)	<0.001
(7) Latest observation or 0.5 kg increase a	57,890	−0.747	(−1.132, −0.361)	<0.001
(8) Latest observation or 1 kg increase a	57,890	−0.711	(−1.112, −0.309)	0.001
(9) Latest observation or 0.5 kg decrease a	57,890	−0.821	(−1.179, −0.464)	<0.001
(10) Latest observation or 1 kg increase a	57,890	−0.860	(−1.206, −0.515)	<0.001

a Model adjusts for age at referral, sex, ethnicity (white/mixed/black/Asian/other), Index of Multiple Deprivation (IMD) quintile, time since baseline (in months) as fixed effects and Clinical Commissioning Group (CCG) nested within Sustainability and Transformation Partnership (STP) as random effects.

Under all assumptions (Table 3), weight change in the digital only cohort was found to be non-inferior to that in face-to-face, like in the main analysis: this is indicated in Table 3 by the upper bound of the confidence intervals being under 1 kg. Under assumptions 1–5, weight change was greater in the digital only cohort than the face-to-face cohort, unlike in the main analysis. However, under assumptions 6–10, which incorporate intermediate weight outcomes, the mean change in weight at 6 months, was non-inferior to face-to-face, but was not superior.

Under all assumptions (Table 4), the estimated mean weight change in the digital choice cohort was non-inferior to that in face-to-face, like in the main analysis: this is indicated in Table 3 by the upper bound of the confidence intervals being under 1 kg. Under all assumptions, after adjustment, the mean change was greater in the digital choice cohort compared to the face-to-face cohort, and this was statistically significant, as in the main analysis. Values of mean weight change were like that in the main analysis for assumptions 1–4 but slightly closer to 0 for assumptions 5–10.

## 4 Discussion

In this exploratory analysis including those who provided a baseline weight measure, missing weight values at 6 months post baseline were imputed, using the last observed weight measure, if available, or assumed plausible outcomes ranging from weight loss of 1 kg to weight gain of 1 kg. Under all ten assumptions, face-to-face, digital only and digital choice diabetes prevention cohorts saw an average reduction in weight at 6 m of between 0.9 kg and 2.2 kg. Under all assumptions, after accounting for differences in baseline characteristics of the three cohorts, weight loss in both digital cohorts was non-inferior to that in the face-to-face cohort. Weight loss in the digital only cohort was like face-to-face, and the difference was no greater than 0.5 kg under any assumption. Weight loss in the digital choice cohort was, on average, superior to face-to-face, between 0.7 kg and 1.1 kg greater. Patients who were offered a choice and chose the digital service achieved significantly more weight loss, compared to patients offered face-to-face only.

These results are consistent with the findings of the primary analysis (Marsden et al., 2022b), which reported that weight change on the digital pilot was non-inferior to face-to-face at 6 months: it was similar in the comparison of those not offered a choice and greater in digital when participants were offered a choice. The similarity with the primary analysis, under all assumptions, provides greater confidence in the finding that digital delivery of a diabetes prevention programme is non inferior to face-to-face delivery. Compared to the primary analysis, which was restricted to those who provided weight measures at 6 months, our analysis included a much larger cohort of people who attended a first session of the face-to-face service or registered for the digital service.

A previous complete case analysis of the NHS digital DPP, including only participants who provided weight measures at baseline and 6 months, reported mean weight loss of 3.5 kg (95% CI: 3.3, 3.7), n = 1,811 (Ross et al., 2022). By comparison, in our analysis, which includes a larger cohort of everyone who registered and provide a baseline weight measure, mean weight loss was between 1.34 and 2.18 kg (digital only, n = 1778) and 0.86 and 2.08 kg (digital choice, 2139). Our results were more conservative than the complete case analysis, as expected when the largest plausible assumption for non-completers was lower than the mean weight loss among completers.

We believe that our approach to missing data is novel among studies of real-world DPPs. In their analysis of the face-to-face DPP, Valabhji et al. performed a complete-case analysis but additionally used multiple imputation in a sensitivity analysis (Valabhji et al., 2020). Using similar data, Marsden et al. also undertook a complete-case analysis, with multiple imputation only for patients who were still participating when the outcomes were collected, assuming the data for others was not missing at random (Marsden et al., 2022a). In Ross et al.’s analysis of the digital DPP, a sensitivity analysis was performed using Inverse Probability Weighting where the observed data was weighted based on the probability of drop-out (Ross et al., 2022). These approaches can be effective for data that is missing at random, but do not remove bias for data missing not at random. They also rely on the relevant models being correctly specified. Ashra et al. performed a *meta*-analysis of RCTs evaluating diabetes prevention programmes and reported that several studies performed complete case analyses, likely inflating the intervention effect sizes (Ashra et al., 2015).

The missing data in this study was disproportionately from among lower-weight, low-income and non-White participants, which is consistent with engagement challenges in prevention services (McGill et al., 2015). Although statistical methods such as this are helpful to the field, it should also be noted that more effort is needed to promote uptake among participants from all backgrounds, especially those at higher risk for diabetes, during delivery of DPPs.

This study has several strengths. The data allows a contemporaneous comparison of face to face and digital delivery of a diabetes prevention service, which has not previously been done. This analysis using plausible assumptions has produced similar results to compete case analysis based on both matching and regression adjustment, suggesting robustness of the findings. Plausible imputation has allowed inclusion of the whole cohort, unlike the complete case approach, and this has permitted something closer to an intent-to-treat analysis, providing greater confidence in the original findings. As with any study based on observational data, the results may be biased by unmeasured confounding.

Our findings that digital can be as effective as face-to-face delivery, and more effective when participants are offered a choice, add to growing evidence that digital delivery has the potential for wider reach at lower cost, and may be more acceptable and accessible to some sectors of the population (Murray et al., 2019; NHS England, 2022). However, digital health interventions can face low rates of uptake and completion (Beleigoli et al., 2019, Murray et al., 2018, Murray et al., 2017).

## 5 Conclusion

Enrolment in the NHS digital diabetes prevention programme was associated with clinically significant reductions in weight which were (at least) equivalent to the weight loss seen in the face-to-face programme. Digital services can be just as effective as face-to-face in delivering a population-based approach to the prevention of type 2 diabetes. Patients who chose digital after being offered a choice of digital or face-to-face achieved (significantly) better weight loss outcomes, compared to patients offered face-to-face only. Indeed, current operational delivery of the Programme now offers participants the choice of face-to-face group-based delivery or digital delivery, as described in the 2022 version of the Programme Service Specification (NHS England, 2022). Imputation of plausible outcomes is a feasible methodological approach, suitable for analysis of routine data in settings where outcomes are systematically missing for non-attenders.

## Ethics committee approval

The research was approved by the North West Greater Manchester East NHS Research Ethics Committee (Reference: 17/NW/0426, 01/08/17, amended 29/09/20).

## Availability of data

The data that support the findings of this study were used under license from NHS England for the current study only and are not publicly available.

## Disclosure of funding

This work is independent research funded by the National Institute for Health and Care Research (Health Services and Delivery Research, 16/48/07 – Evaluating the NHS Diabetes Prevention Programme (NHS DPP): the DIPLOMA research programme (Diabetes Prevention – Long Term Multimethod Assessment)). The views and opinions expressed in this manuscript are those of the authors and do not necessarily reflect those of the National Institute for Health and Care Research or the Department of Health and Social Care.

## CRediT authorship contribution statement

**Antonia M. Marsden:** Methodology, Validation, Formal analysis, Data curation, Project administration, Investigation, Writing – original draft. **Mark Hann:** Methodology, Validation, Investigation, Writing – review & editing. **Emma Barron:** Resources, Writing – review & editing. **Jamie Ross:** Resources, Writing – review & editing. **Jonathan Valabhji:** Conceptualization, Writing – review & editing. **Elizabeth Murray:** Conceptualization, Funding acquisition, Writing – review & editing. **Sarah Cotterill:** Conceptualization, Funding acquisition, Supervision, Methodology, Writing – original draft.

## Declaration of Competing Interest

AM, MH, EB, BM and SC declare that they have no known competing financial interests or personal relationships that could have appeared to influence the work reported in this paper. EM is managing director of a not-for-profit Community Interest Company, HeLP-Digital, which exists to disseminate a digital diabetes self-management programme, HeLP-Diabetes, across the NHS. JV is the national clinical director for diabetes and obesity at NHS England.
